# Occurrence and role of lung megakaryocytes in infection and inflammation

**DOI:** 10.3389/fimmu.2022.1029223

**Published:** 2022-11-29

**Authors:** Lucie Gelon, Léa Fromont, Emma Lefrançais

**Affiliations:** Institut de Pharmacologie et de Biologie Structurale (IPBS), Université de Toulouse, CNRS, Université Toulouse III - Paul Sabatier (UPS), Toulouse, France

**Keywords:** lung megakaryocyte, platelet, emergency megakaryopoiesis, infection, inflammation

## Abstract

Megakaryocytes (MKs) are large cells giving rise to platelets. It is well established that in adults, MKs develop from hematopoietic stem cells and reside in the bone marrow. MKs are also rare but normal constituents of the venous blood returning to the lungs, and MKs are found in the lung vasculature (MK_circ_), suggesting that these cells are migrants from the bone marrow and get trapped in lung capillaries where the final steps of platelet production can occur. An unprecedented increase in the number of lung and circulating MKs was described in coronavirus disease 2019 (COVID-19) patients, suggesting that lung thrombopoiesis may be increased during lung infection and/or thromboinflammation. In addition to the population of platelet-producing intravascular MKs in the lung, a population of lung-resident megakaryocytes (MK_L_) has been identified and presents a specific immune signature compared to its bone marrow counterparts. Recent single-cell analysis and intravital imaging have helped us gain a better understanding of these populations in mouse and human. This review aims at summarizing the recent data on increased occurrence of lung MKs and discusses their origin, specificities, and potential role in homeostasis and inflammatory and infectious lung diseases. Here, we address remaining questions, controversies, and methodologic challenges for further studies of both MK_circ_ and MK_L_.

## Introduction

Platelets are small anucleate blood cells with active roles in a wide range of physiological responses, including hemostasis, thrombosis, wound healing, and immunity. In humans, 150–450 billion platelets circulate per liter of blood, with a life span of 8–10 days, requiring a daily turnover of 100 billion platelets (70 million/min) to maintain a physiological platelet concentration. Platelets originate from megakaryocytes (MKs), large cells described for the first time in 1890 by Howell and Donahue (1) . With an approximate diameter of 100 μm, MKs are large but rare cells, accounting for 10 MK/ml in venous blood and representing only 0.01% of all nucleated cells in human bone marrow. This paucity, as well as the importance of their *in vivo* environment for their maintenance, differentiation, and platelet production, makes it difficult to isolate and study them. Thus, the understanding of MK biology still contains some gray areas compared to other hematopoietic cell types. Recently, the development of single-cell analysis and *in vivo* analysis have revealed new insights into the MK journey from progenitor cells to platelets, particularly regarding the mechanisms of differentiation, their heterogeneity, the final steps of platelet generation, and the different locations involved. The relative importance of lung thrombopoiesis and the location, occurrence, origin, and function of lung MKs in homeostasis and during infection and inflammation will be discussed in this review.

## Sites of megakaryocyte maturation and platelet production

**Figure 1:**
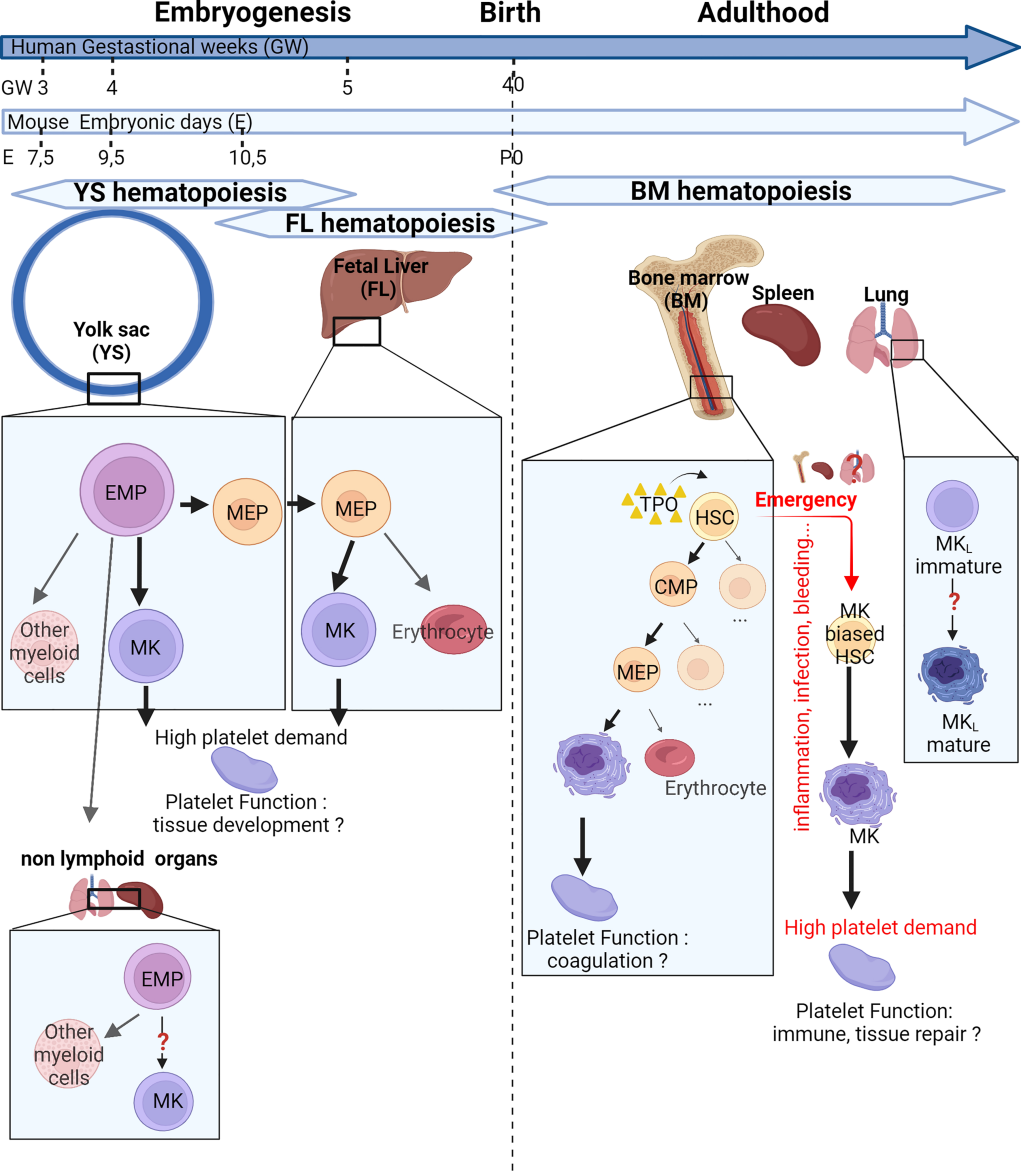
Place and mechanisms of megakaryocyte differentiation over time. The hematopoiesis begins in the yolk sac at 7.5 days post-conception (E7.5) in mice or 3 gestational weeks (GW) in human. At E9.5 or at GW4, the first megakaryocytes (MKs) originate from yolk sac-derived erythroid–myeloid progenitors (EMPs) bypassing the bipotential MK–erythrocyte progenitors (MEPs). Later, a new wave of MKs originate from bipotential MEPs in the fetal liver. EMPs can colonize nonlymphoid organs and are capable of differentiation in myeloid cells. After birth, the bone marrow gradually takes over from the liver. MKs can differentiate from multipotent progenitors (MPPs) or, in case of high platelet need, directly from MK-biased hematopoietic stem cells (HSCs). Extramedullary megakaryopoiesis also occurs in the spleen in case of sepsis. Finally, the lung presents MK progenitors and resident MKs presenting an immune phenotype. These different MK subsets could produce specialized platelet populations capable of immune functions and tissue development. CMP, common myeloid progenitor; MKL, Lung resident Megakaryocytes; TPO, Thrombopoietin.

**Figure 2:**
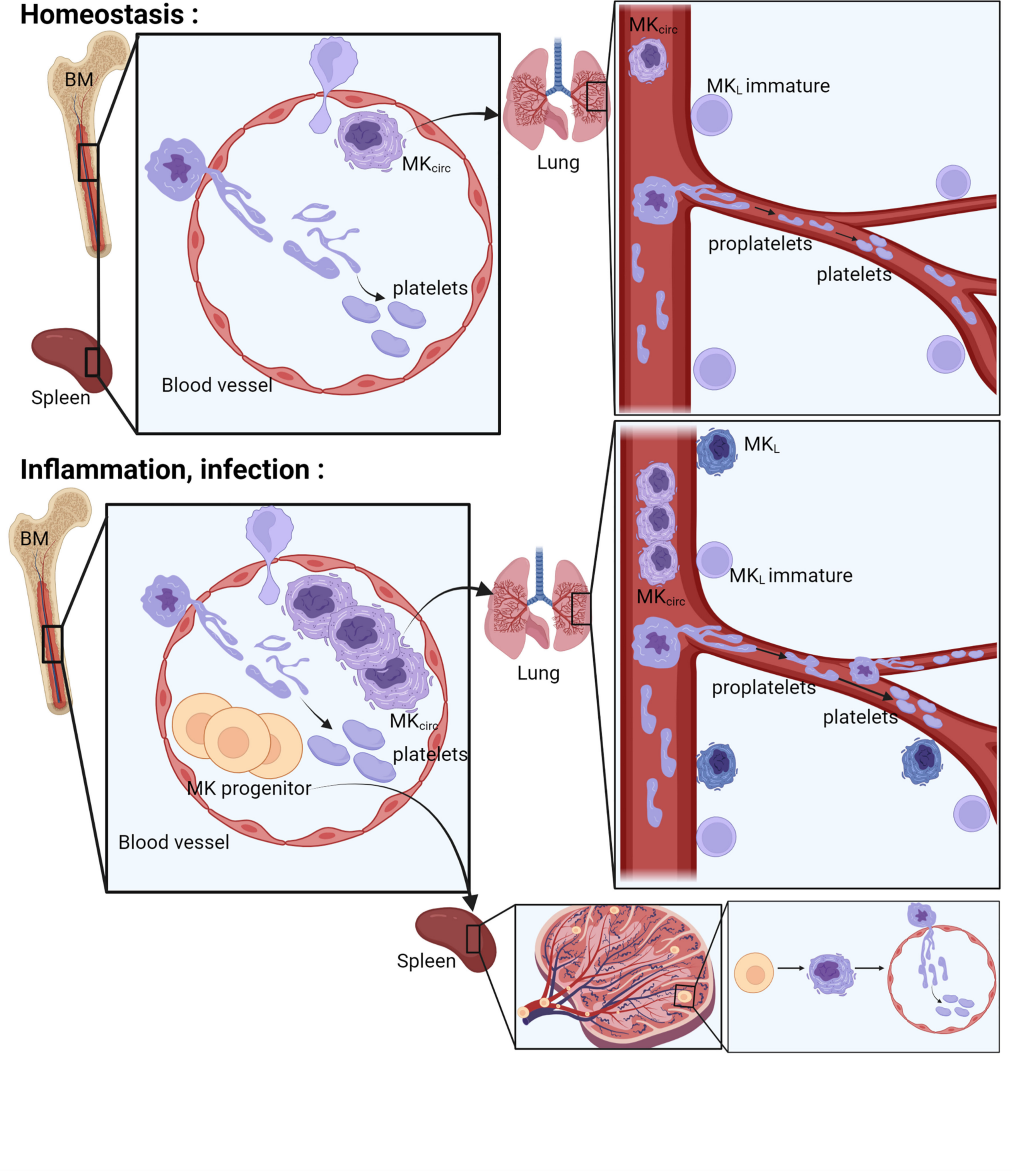
Place of platelet production. The bone marrow, the spleen, and the lung have been described as places where the final steps of platelet production occur. In the bone marrow and in the spleen, the MKs extend their cytoplasm to the lumen of the sinusoids and form proplatelets. Proplatelets and more rarely whole MKs are released in the bloodstream, get trapped in the lung vasculature where they will accomplish the final step of platelet production. During inflammation or infection, an increase of circulating MKs and MK progenitors is observed, followed by progenitor differentiation and platelet production in the spleen and increased platelet production in the lung vasculature. MK, megakaryocyte; MKL, Lung resident Megakaryocytes; MKcirc, Circulating Megakaryocytes; BM, Bone Marrow.

Thrombopoiesis, the process of platelet production, can be separated into two steps: the differentiation and maturation of MKs (megakaryopoiesis) (**Figure 1**), followed by the actual process of platelet release resulting from MK cytoplasmic extensions called proplatelets (**Figure 2**). These two steps can take place in different sites, in the bone marrow and outside, evolving and adapting to platelet needs during development, aging, or stress.

### Pathways of megakaryocyte differentiation

Megakaryopoiesis has long been described as a hierarchical process, with hematopoietic stem cells (HSCs) giving rise to various blood lineages including the common lymphoid progenitor (CLP) and the common myeloid progenitor (CMP). The CMP further differentiates into the granulocyte/macrophage progenitor and the megakaryocyte–erythrocyte progenitor (MEP), which then further differentiate into MK progenitors and ultimately into mature MKs. Over the last few years, studies have challenged traditional paradigms about the origin of the MK lineage in both fetal and adult life, and the application of single-cell RNA sequencing (RNA-Seq) has led to a better characterization of embryonic, fetal, and adult MKs. The hierarchical model of MK differentiation was challenged by the observations of MK-biased hematopoietic progenitors and alternative routes by which MKs can directly differentiate from HSCs, bypassing traditional lineage checkpoints and progenitors (2–4). The production of platelets from MK-biased hematopoietic progenitors is observed preferentially after reduced platelet count (5), inflammation, myeloproliferative neoplasms, aging, and recently, respiratory viral infection (6), also called “emergency” megakaryopoiesis and reviewed by Noetzli et al. (7) (**Figure 1**). Mechanistically, MK-biased HSC is posttranscriptionally regulated by the repression of MK-specific transcript translation steady state (8) and before birth (9). The restriction, maintained by the RNA-binding protein LIN28B, is lifted during development or inflammatory stress by the downregulation of the protein, leading to the increase of MK-biased HSCs.

### Places of megakaryocyte differentiation

During mouse embryogenesis, two pathways have recently been uncovered to produce definitive MKs (**Figure 1**). The initial source of platelets comes from the yolk sac and starts at Embryonic day (E) 9.5 directly from yolk sac-derived erythroid-myeloid progenitors (EMPs), the earliest precursors of erythrocytes, MKs, and macrophages, bypassing intermediate bipotent MEPs (10, 11). Later in development, yolk sac-EMPs give rise to bipotent MEPs migrating to the fetal liver starting from E10.5 and terminally differentiate into both erythrocytes and polyploid MKs in the fetal liver (10). In human, a shared megakaryocyte–erythroid–mast cell progenitor (MEMP) was identified in fetal liver, also present in non-lymphoid tissues (skin and kidney) from 7 to 12 weeks after conception. HSC/multipotent progenitors (MPPs) were absent in non-lymphoid tissues where MEMPs can locally differentiate (12). A few months after birth, the contribution of the fetal liver as a main site of platelet production declines progressively and the bone marrow takes over platelet production, lasting through adulthood. Other sites of hematopoiesis and megakaryopoiesis outside of the bone marrow have been described, particularly in response to hematopoietic stress, severe infection, and inflammation. MKs and progenitors are present in healthy spleen in mouse and human (13), where increased MK maturation has been observed after murine HSC transplantation (14, 15), sepsis (13), or myelofibrosis (16). Recent reports in the literature also indicate the presence of hematopoietic stem and progenitor cells (17, 18) and in particular MKs in fetal and adult lung, with a transcriptional signature suggesting a role in immunity [lung-resident megakaryocytes (MK_L_)] (19, 20). An idea of the relative presence of MKs in different locations Bone marrow (BM), spleen, and lung] is given by the quantification of MKs by flow cytometry in the adult mouse, indicating that 79% of MKs are found in the BM, 13% in the spleen, and 8% in the lungs (without exclusion of circulating MKs and cells aggregated with platelets) (21).

### Places of final platelet production

In the final phases of differentiation, MKs, residing in close proximity to BM sinusoids (22), interact with endothelial cells and, using a transcellular route, extend protrusive podosomes to elongate their cytoplasm to form branched pseudopodial extensions called proplatelets (23) (**Figure 2**). These proplatelets extend into the lumen of bone marrow sinusoids and, under shear and turbulent flow, are released into the bloodstream. The final release of platelets then occurs within the blood where a particular role for the lung vasculature has been proposed. Indeed, human MKs transfused into mice end up trapped in the lung vasculature and release functional platelets (with the ability to incorporate into developing thrombi after laser-induced injuries) (24, 25). Proplatelets and entire MKs have been observed in the lung vasculature since 1930s (1, 26–28), and abundant cytoplasm-circulating MKs are found in higher number in the blood that enter the lung than in the blood exiting it. Thanks to the development of intravital microscopy technique to the lung, and as an ultimate proof of platelet release in the lung, direct and dynamic platelet production was observed in mouse lung vasculature (19). MKs are observed in the lung vasculature physiologically (MK_circ_), but their frequency increases with pathological conditions, likely associated with an increased platelet production following platelet consumption observed during lung infection and/or thromboinflammation, as largely reported in coronavirus disease 2019 (COVID-19). This increased occurrence will be reviewed specifically in a following section.

## Location of megakaryocytes in the lung of healthy individuals

### Circulating megakaryocytes found in lung capillaries (MK_circ_)

We can distinguish two types of lung MKs: those found in the vasculature (MK_circ_) and those found residing in the tissue (MK_L_). In healthy human, most of the large MKs, recognized by the size of their cytoplasm and nucleus, are intravascular, localized within the capillary lumen of the alveolar wall. They are found in histological sections in 67% of healthy autopsy (individuals who died suddenly and without any injury of the thorax), with an average value of 4 MK/cm^2^ (maximum of 16 MK/cm^2^) of lung tissue (26, 29, 30). Most of the MK_circ_ (75%) are medium sized (20–49 µm; with a moderate amount of cytoplasm), 10% are large MKs (50–100 µm; with abundant cytoplasm), and 15% are small MKs (<20 µm; with a thin rim of cytoplasm, resembling naked or semi-naked nuclei) (28). MK_circ_ can also be quantified from the venous blood, before entering the lung. An average value of 10 MK/ml (maximum of 30 MK/ml) is detected, half of them with abundant cytoplasm and the numbers are markedly reduced in the arterial circulation (after lung filtration) with the majority of MKs devoid of cytoplasm (naked MKs). With the development of intravital microscopy, direct observation of dynamic events occurring in mouse lungs has been made possible (31). Using platelet/MK reporter mice, direct evidence was provided that platelet release occurs physiologically in the lung (19). Both nucleated MKs and large cytoplasmic fragments are observed in the pulmonary circulation, trapped at vascular bifurcations. MKs and proplatelets are clearly differentiated from platelet aggregates and thrombus that can occur in lung vessels following platelet activation (32). During a process ranging from 20 to 60 min, proplatelet extension results in the release of 150–2,500 single platelets (average of 700). In the subpleural region observed by lung intravital microscopy (2 mm^2^ × 40 µm), two MKs/proplatelets per hour were witnessed releasing platelets in the lung vasculature. Altogether, these reports indicate that platelet release occurs physiologically in the lung vasculature from circulating MKs. The contribution of the lung in platelet production has been a matter of debate, as a study indicates that in lung tissue, MKs were rare in whole cross sections from normal and perturbed mice, even during periods of strong stimulation of thrombopoiesis (33). Lung MKs are not uniformly distributed in the lung and are preferentially localized to the distal lung within the interstitial space or within the capillary lumen between adjacent alveoli (28) (34) but not deeper in the lung (33). The alveolar region is also the region accessible by intravital microscopy where platelet release in the lung was observed (19). Morphologically, MK_circ_ are squeezed within alveolar capillaries and will be harder to identify than resident MKs in the BM or spleen. Attempts were made and reviewed to estimate the contribution of the lung in the terminal step of platelet production (35–38). All of them propose a significant contribution of the lung, ranging from 7% to 98%, even though these calculations have weaknesses such as the use of approximated values for platelet life span, percentage of circulating MK reaching the lung, percentage of platelets sequestrated in the spleen, platelets produced per MK, or extrapolation of observed region to whole lung. Interestingly, thrombocytopenia is common in patients with congenital heart conditions associated with right to left shunting and following cardiopulmonary bypass, with an incidence over 30%. Two to three days following surgery, the average platelet count decreases by half. It is classically attributed to consumption of platelets activated during the extracorporeal circulation. However, an intriguing finding is that MK_circ_ increase 3-fold in these patients who have the lung circulation bypassed. Thrombocytopenia in these settings could be due to underproduction of platelets in the lung, in addition to consumption (39). One can wonder how these large cells (~50 µm) can pass through such small capillaries (5 µm). *In vitro*, in a circulatory flow system, it was shown that mature MKs can divide in the circulation. The MK deforms and elongates, and the nucleus tends to separate into two distinct lobes in parallel with proplatelet formation. Intact large MKs have been observed after lung filtration, suggesting that they can go through the capillary beds without fragmentation (40). The deformation occurring *in vivo* in the lung capillaries and its impact on the MK biology remain to be elucidated.

### Lung-resident megakaryocytes in the interstitium (MK_L_)

In addition to the population of platelet-producing intravascular MKs in the mouse lung (MK_circ_), a second population of MKs, residing in the pulmonary interstitium, has been identified in the mouse lung (MK_L_)—thanks to their expression of specific MK markers by three independent studies and were further analyzed by bulk or single-cell RNA-Seq analysis (19, 20, 34). MK_L_ represent 0.1% to 2%–3% of lung cells. Different markers were used to identify the MK_L_ population: Lin^-^, CD41^+^, PF4-expressing nucleated cells (19); Lin^-^CD41^+^ cells; or clustering based on *Pf4*, *Itga2b*, *Ppbp*, *Itgb3*, *Gp9*, and *Cxcr4* (20) or isolation of top 1% of CD41^+^ events and MK-lineage clustering based on *Fli1*, *Pf4*, and *Itga2b* (34). Contrary to human observations of lung tissue section where most of lung MKs were intravascular, resident MK_L_ actually represent the majority of lung MKs (60%–80%). This discrepancy may come from the fact that MK_L_ are smaller and more immature than MKs present in the bone marrow or those found in the vasculature (MK_circ_). MK_L_ are mainly diploid and may not have been included in the human studies that identified MK by the large size of their cytoplasm and nucleus. Yeung et al. (34) did not find MK_L_ to be immature, but it may be explained by their isolation strategy using only the top 1% of CD41^+^ events, thus focusing the analysis on more mature MKs. A common result from these three studies is the differential expression of many genes associated with innate immunity, inflammation, and pathogen recognition compared with bone marrow MKs, pointing to a potential immune role for lung extravascular MKs that will be further discussed.

## Modulation of lung megakaryocyte occurrence with pathologies

### Increased presence of megakaryocytes in the lung (MK_L_ and MK_circ_)

We have seen that MKs are found in 67% of healthy lungs (4 MK/cm^2^), but they are almost always found in 94% of hospitalized patients and in higher density with an average value of 37 MK/cm^2^ (maximum of 765 MK/cm^2^) (26, 29), suggesting an increased occurrence with various pathologies. Pathologies associated with an increase or decrease of lung MKs are listed in **Table 1**. In human, lung MKs were increased in association with vascular diseases often associated with excessive coagulation (thromboembolic disease, intravascular coagulation, myocardial infarction, and severe atheroma) (26, 28, 29), tissue damage and bleeding (shock, burns (41), hemorrhage), cancer (42, 43), inflammatory lung diseases [acute respiratory distress syndrome (ARDS) (44), idiopathic pulmonary arterial hypertension (28), fibrosis, asthma (45)], and infectious diseases. More recently, the COVID-19 pandemic has particularly revealed the presence of MKs in the lungs of patients. In fact, between 7 and 10 times more MKs have been counted in the lungs of patients with COVID-19 compared to healthy patients and between 2 and 3 times more compared to patients with other causes of ARDS (46–48). MKs found in COVID-19 non-survivor patients show signs of maturation (increased diameter and bigger nucleus) and are found 2.5 times more frequently in the alveoli/parenchyma than in the blood vessel. The majority of lung MKs (70%) are found outside of the vasculature in the lung. The increase in MK_L_ is associated with an equal increase in the bronchoalveolar lavage (BAL), in the BM, and in the circulation of these patients (49). MKs entrapped in lung vasculature were also observed after influenza infection (50). In healthy lungs, MKs are not observed in alveolar or pleural fluid, but they were detected in human BAL of patients with severe COVID-19 (49) and suspected lung carcinoma (51) and in pleural fluids in patients with myeloproliferative disorders, trauma, and tumors. In those cases, the presence of MK_L_ may be the result of increased vascular and epithelial permeability, inducing MK_circ_ entry in the tissue and fluid or the result of extramedullar hematopoiesis (52). The common pathogenic mechanism explaining the increased number of pulmonary MKs seems thus to be associated with platelet loss due to consumption (due to activation in case of excessive coagulation or inflammation) or bleeding and may be associated with accelerated thrombopoietic activity. In animal models, an increase in pulmonary MKs was also observed following *Listeria monocytogenes* infection (53), thrombin-induced microthrombi in dogs (54), systemic inflammation induced by Tumor necrosis factor alpha (TNF-α) in rats (55), bleomycin-induced lung damage and fibrosis (56), Thrombopoietin (TPO)-accelerated thrombopoiesis (19, 27), and bleeding (27) in mice.

**Table 1:** Increased and decreased occurrence of lung megakaryocytes with pathologies.

Class of disease	Reference	Disease	Species	Increase/Decrease	Measures	Specificities
**Cardiovascular** **Disease**	Sharma & Talbot, 1986	Severe coronary atheroma	Human	1.8×	Histology	No significant increase in mild and moderate coronary atheroma. Similar increase of pulmonary MK in cerebral and aortic atheroma.
	Sharma & Talbot, 1986	Myocardial infarct	Human	1.8×	Histology	Increased mean platelet volume. Might be caused by hypoxia, atherothrombosis, or disturbance of the lung and MK interaction
	Aabo & Hansen, 1978; Wells et al., 1984	Microthrombosis/DIC	Human	↗	Histology	The thrombosis are found in burns (Wells et al., 1984) and cancer (Aabo & Hansen, 1978).
**Tissue damage and bleedings**	Aabo & Hansen, 1978; Sharma & Talbot, 1986	Shock	Human	1.7×	Histology	Only slight increase in thromboembolic disease
	Aabo & Hansen, 1978; Zucker-Franklin & Philipp, 2000	Hemorrhages	Human	↗	Histology	Cases with major hemorrhages all had more than 50 MK/cm²
	Aabo & Hansen, 1978	Liver insufficiency	Human	↗	Histology	Mostly cirrhosis and metastases with icterus
**Cancer**	Aabo & Hansen, 1978; Sharma & Talbot, 1986; Hume, et al., 1964	Cancer (Pelvic, Head, and Neck)	Human	50× (antecubital vein MK_circ_)	Histology and Circulation	MK_circ_ are taken in the antecubital vein blood and the regional venous blood. They occur more frequently in the regional venous blood. There is a diurnal pattern in the occurrence.
	Soares, et al., 1992	Lung cancer	Human	5× compared to healthy lung	Histology	Only with the presence of tumor emboli or lung metastases.
**Inflammatory lung disease**	Mandal, Mark, & Kradin, 2007; Rapkiewicz et al., 2020	ARDS	Human	5–10× compared to normal lung	Histology	Greater number of CD61+ MK in ARDS and even greater in drug toxicity-induced ARDS.
	J. Balko, et al., 2022	Idiopathic pulmonary arterial hypertension	Human	9× compared to normal lung	Histology	Donors from the lung transplantation program. Morphological features of MK (clustering, cytomorphology).
	Slater D, Martin, Trowbridge A., 1985	Asthma	Human	↗	Histology	
	Sharma & Talbot, 1986	Smoking	Human	1.5× with smoking history	Histology	** **
**Infectious diseases**	Aabo & Hansen, 1978; Sharma & Talbot, 1986; Wang et al., 2021	Acute infection	Human/Mice	1.3–3×	Histology	Mostly pneumonia.
	Rapkiewicz et al., 2020; Roncati et al., 2020; Valdivia-Mazeyra et al., 2021; Zhu et al., 2022	COVID-19	Human	7–10× compared to healthy donor. 2–3× compared to other ARDS.	Histology and circulation	The increase in lung is associated with an increase in BAL, BM, and PBMCs. Circulating MK with unique transcriptome. MKs and platelets are infected by SARS-CoV-2. Emergency megakaryopoiesis.
**Induced lung damage in animals**	Warheit et al., 1989	Thrombin-induced microthrombi	Dog	↗	Histology and circulation	75% of the arterial MK_circ_ were devoid of cytoplasm, and 30% of venous MK_circ_ consisted of naked nuclei. Migration from the BM
	Sulkowski, Terlikowski, & Sulkowska, 1999	Lung inflammation TNF-alpha	Rat	↗	Histology	Occlusion of vessels by megakaryocytes. Common naked nuclei or MK cytoplasm fragmented.
	Zhou, et al., 2019	Bleomycin-induced lung fibrosis	Mice	8× compared to untreated mice	Histology	MK promotes the proliferation of primary lung fibroblasts and transdifferentiation to myofibroblasts.
	Lefrancais,2017; Zucker-Franklin & Philipp, 2000	TPO-accelerated thrombopoiesis	Mice	2× compared to untreated mice	Histology and LIVM	Phlebotomized or TPO treatment.
**Thrombo-cytopenia**	Aabo & Hansen, 1978	Thrombocytopenia	Human	↘	Histology	Depression of the bone marrow will result in a decreased number of circulating MKs such as in leukemia.
**COPD**	J. Balko, et al., 2022	COPD	Human	Decrease of 2×	Histology	Isolated MKs inside capillaries of interalveolar septa.
**Cancer**	Aabo & Hansen, 1978	Leukemia	Human	↘	Histology	Leukemia was found in 18 cases, and none of them had an increased number of pulmonary MKs.
**Hypoxia**	Wu et al., 2021	Hypoxia	Mice	Decrease of 1.6×	Flow, IF	Reduced BM output. Reduced platelet production. Impair thrombocytopoiesis in the lung.

ARDS, acute respiratory distress syndrome; BAL, Bronchoalveolar Lavage; CD, cluster of differentiation; COPD, Chronic obstructive pulmonary disease; COVID-19, Coronavirus Disease 2019; BM, Bone marrow; DIC, Disseminated Intravascular Coagulation; IF, Immunofluorescence; LIVM, Lung intravital microscopy; MK, megakaryocyte; MKcirc, Circulating MK; PBMC, peripheral blood mononuclear cell; SARS-CoV2, Severe acute respiratory syndrome coronavirus 2; TNF, Tumor necrosis factor; TPO, thrombopoietin.

↗, increased occurence of lung megakaryocytes; ↘, decrease occurence of lung megakaryocytes.

### Decreased presence of lung megakaryocytes

A reduced number of pulmonary MKs are less often described, but in human, low values were correlated with thrombocytopenia, leukemia (29), and Chronic obstructive pulmonary disease (COPD) (28). In mouse, a decrease was observed in the lung of hypoxic mice, associated with reduced platelet production (57). *In vitro* hypoxia enhances human lymphoid differentiation (58) and biased hematopoiesis toward lymphocyte generation or reduced thrombopoiesis may explain the decreased number of pulmonary MK.

## Origin of circulating megakaryocytes

### MK/proplatelet egress from extrapulmonary sources (bone marrow, spleen)

Platelet-producing MKs and proplatelets found in the lung circulation are thought to come from other organs such as the bone marrow and spleen. Indeed, by examining mouse lungs with non-fluorescent MK/platelet transplanted into MK/platelet fluorescent mice, fluorescent MKs were observed in the lung vasculature, while no MK_circ_ was observed in fluorescent MK/platelet lung transplanted into MK/platelet non-fluorescent mice. This means that the production of platelets in the lung circulation is dependent on MKs exiting the bone marrow or other extrapulmonary sources and traveling to the lung. Direct visualization of the bone marrow and spleen confirms the release of proplatelets exceeding platelet size into marrow sinusoids (23, 59–61). In most cases, proplatelets are released, but evidence of entire MKs leaving the bone marrow entering the circulation has been visualized, sometimes extruded from the BM attached to the released proplatelet extensions (19, 21, 22, 62, 63). The rare frequency of entire MKs in the lumen of the BM sinusoids (representing 1%–2% of all BM MKs) has been advanced to challenge the model whereby MKs emerge from the bone marrow and are trapped in the lung vasculature. MK_circ_ are indeed rare events (10 MK/ml), and *in vivo*, the majority of platelets released in the lung vasculature originate from large cytoplasmic fragments or proplatelets and ~15% are released from whole nucleated MKs (19). Considering that the lung vasculature carries the blood from the whole body, it is expected that the chance of observing entire MKs extruding from the BM will be lower than the observation of entire MKs releasing platelets in the lung vasculature. All bones are also not equal in their hematopoietic cell contents, and the sites of blood formation evolve with age, gradually shifting to the vertebrae, sternum, ribs, and pelvic bones. MK egress may occur preferentially in specific regions. The MK_circ_ could also come from other extrapulmonary sources such as the spleen, as maturation and large proplatelet extension have been observed in mouse and human (13, 19).

### Mechanisms of MK/proplatelet egress

**Figure 3:**
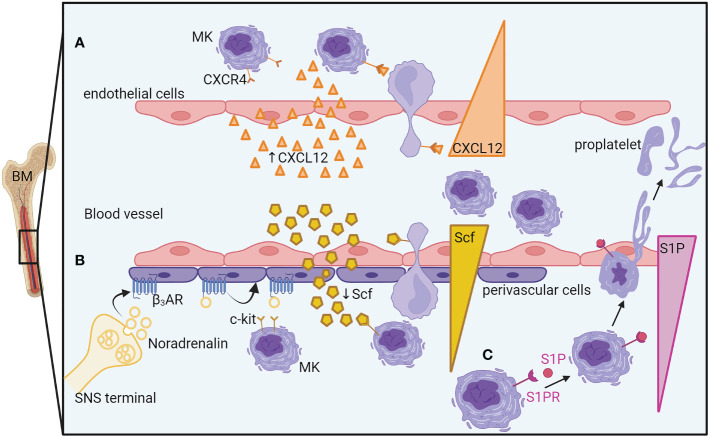
Mechanisms of megakaryocyte egress and proplatelet release from the bone marrow. **(A)** An increase of endothelial cell-derived CXCL12 in the blood guides the MK, thanks to its CXCL12 receptor, CXCR4, close to the sinusoidal vascular niche where it would deform itself to enter the vasculature. **(B)** During sepsis, the sympathetic nervous system releases noradrenaline, inducing the reduction of perivascular cell-produced SCF in the bone marrow. The SCF gradient between the bone marrow and the blood will guide the MKs to exit in the blood vessels. **(C)** A higher concentration of S1P in the blood than in the bone marrow guides MK elongation into the bloodstream by S1PR activation. BM, Bone marrow; MK, megakaryocyte; CXCR4, C-X-C chemokine receptor type 4; CXCL12/SDF1, C-X-C motif chemokine 12/ stromal cell-derived factor 1; SCF, Stem cell factor; c-kit/SCFR, Stem cell factor receptor; β3-AR, beta-3 adrenergic receptor; SNS, Sympathetic Nervous System; S1P, Sphingosine-1-phosphate; S1PR, S1P receptor.

The production of platelets in the lung circulation is dependent on MKs and proplatelets exiting the bone marrow, but the mechanisms of egress need to be better understood. Thin proplatelets, thick proplatelets, or entire MKs can be released from the BM into the circulation, the last two being more likely to be trapped in the lung vasculature (60, 62, 64). If the thin proplatelets predominate in the physiological state, the large proplatelets, more immature, are more frequently observed in case of high platelet demand (acute thrombocytopenia, bleeding, inflammation). Other mechanisms besides proplatelet generation have been proposed such as MK rupture (60) and budding (21), even though these mechanisms were not observed by other groups (22), even in cases of acute platelet need. The mechanisms that have been recently proposed for regulating MK and proplatelet release are depicted in **Figure 3**. The first one involves the chemokine stromal cell-derived factor-1 (SDF-1/CXCL12), an important regulator of MK guidance to the sinusoidal vascular niche. In a mouse model of bleomycin-induced fibrosis, treatment with a specific inhibitor of CXCL12/CXCR4 axis was sufficient to prevent the migration of MKs to lung tissue *ex vivo* and *in vivo* (56). Confirming an important role of CXCL12/CXCR4 signaling in BM MK egress, another study showed that a subpopulation of MKs (CXCR4^high^ MKs) leaves the BM after *L. monocytogenes* bacterial infection. Here, the infection decreased the expression of CXCL12 in bone marrow but increased its expression in the lung, liver, and spleen, suggesting that the different CXCL12 levels between tissues might drive the migration of CXCR4^high^ MKs. In a sepsis model, however, where MKs are maturing in the spleen, inhibiting the CXCL12 pathway did not have a major role in MK egress from the BM. In that case, the release of MK progenitors from the BM was mediated by increased SCF (stem cell factor or kit-ligand) level in the blood and reduced SCF level in the BM. The decreased expression of SCF in LepR+ perivascular cells was regulated by the sympathetic nervous system (SNS) activity acting on β3-adrenergic receptors present on the perivascular cells (13). Another important pathway for transendothelial proplatelet migration and proplatelet shedding into the circulating blood includes the sphingosine-1 phosphate (S1P). S1P has been shown as an important regulator of proplatelet release. Higher concentrations of S1P are found in the blood compared with the bone marrow interstitium. S1P activates Sphingosine-1-phosphate receptor (S1PR) on MKs, guiding proplatelet elongation and shedding into the bloodstream (61). A gradient-independent mechanism has also been described where S1P release in the BM decreases thrombopoiesis and platelet release in the bloodstream (65).

### MK_circ_ and emergency megakaryopoiesis

Single-cell analysis of circulating cells in COVID-19 has supported the hypothesis of a particular pathway of differentiation for MK_circ_. The increased circulating MKs observed in COVID-19 are associated with a specific transcriptome and are enriched in genes associated with shortened MK differentiation process, inflammation, and viral sensing by Interferon (IFN)-stimulated genes (49, 66). Circulating and BM cells present altered hematopoiesis with MK-biased gene expression in the earliest CD34^+^CD38^+^ Hematopoietic stem and progenitor cells (HSPCs) and expanded MK progenitors in response to COVID-19, suggestive of inflammation-induced emergency megakaryopoiesis (67, 68). In Severe acute respiratory syndrome coronavirus 2 (SARS-CoV-2) infection, platelet consumption induced by the formation of microthrombi or by pulmonary inflammation occurs. Increased thrombopoiesis compensates for this platelet loss, resulting in a slight or no reduction in platelet count. High immature platelet fraction and increased mean platelet volume found in patients with COVID-19 support the accelerated rate of platelet turnover (69). These data from COVID-19 patients confirm the association between acute platelet need, increased thrombopoiesis, emergency megakaryopoiesis, and increased MK_circ_. Increased MK_circ_ suggest a subsequent increase in lung platelet production even though dynamic visualization will be needed to observe if the MK_circ_ found in the lung vasculature in COVID-19 or during other inflammatory/infectious diseases are actually producing platelets or if the transit of MK_circ_ through the lung is perturbed, being trapped in the damaged/inflamed lung vasculature.

## The lung vasculature—The ideal place for efficient platelet production?

**Figure 4:**
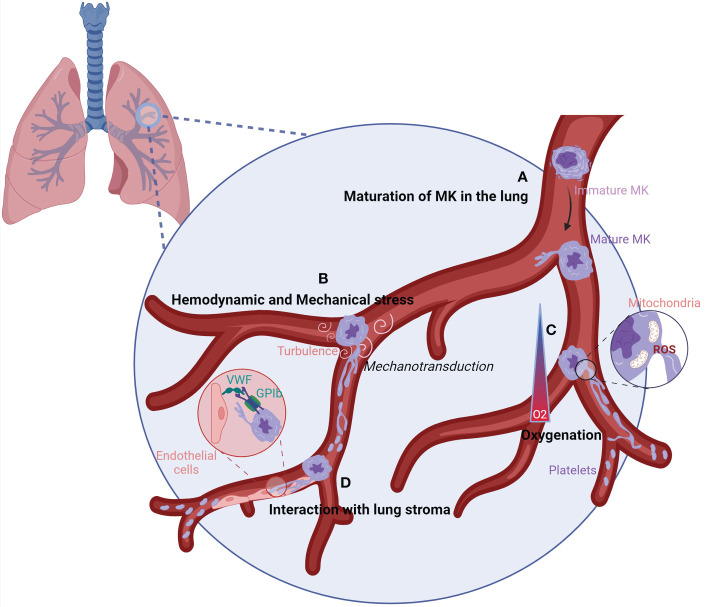
Characteristics of the lung vasculature that could favor efficient platelet production.**(A)** When MKs reach the lung vasculature, they can sense the lung microenvironment leading to their maturation into adaptated MKs. **(B)** The lung vasculature could harbor the perfect size and turbulence for MK elongation into platelets. Following sensing of mechanical stress and high turbulence by MKs, signals could be transmitted by mechanotransduction leading to proplatelet and platelet production. **(C)** The oxygenation of the lung vasculature is elevated, which could increase the mitochondrial fission and generation of mtROS at the MK membrane. ROS production induces the remodeling of the cytoskeleton, ending up in platelet production. **(D)** The interaction between vWF, enriched on lung endothelial cells, and GPIb on MKs could allow MKs to sense shear stress, resulting in the elongation of MKs and platelet formation. GP1b/CD42, Glycoprotein 1b/Cluster of Differenciation 42; MK, megakaryocyte; O2, Oxygen; ROS, Reactive Oxygen Species; vWF, von Willebrand factor.

Currently, *in vitro* platelet production systems failed to reproduce platelet biology and to achieve the yield obtained in the lung when MKs are injected into the circulation *ex vivo* (40) or *in vivo* (19, 24). The lung presents the largest capillary network, in which the hemodynamic forces (blood flow irregular fluctuation, shear stress), oxygenation, and interactions with the stroma could be ideal for platelet production (**Figure 4**).

### Hemodynamic and mechanical stress

The large size of MKs naturally obstructs transit through the lung, which can have capillaries that are <5 µm in diameter. The size exclusion of MKs will induce mechanical stress. MKs are able to sense physical stress, and the activation of mechanotransduction pathways promotes activation and maturation of MKs, reorganization of the cytoskeleton, and generation of proplatelets (70–72). Increasing *in vitro* (73, 74) and *in vivo* evidence (75) suggests that blood flow-dependent shear stress and turbulence are crucial for platelet biogenesis. *In vitro*, final platelet release is best reproduced at high shear rates, superior to the shear rate at the sinusoid wall in the bone marrow. By coupling skull intravital imaging with particle image velocimetry, Ito et al. showed that MKs displaying proplatelet protrusion and platelet release were adjacent to areas of high vascular turbulence. In contrast, resting MKs that did not release platelets were exposed to continuous laminar flow with no turbulence. Pulmonary vessels, the first capillary bed encountered by MKs and proplatelets leaving the bone marrow, may provide an optimal level of turbulence and shear stress required for efficient platelet generation. Corroborating this hypothesis, murine and human MKs grown *in vitro* and injected intravenously are trapped in mouse lungs and shed platelets with normal size distribution, circulating half-life, and functionality (24, 25).

### Oxygenation

The lung vasculature harbors a high level of oxygen compared to the bone marrow, which has recently been revealed as an important parameter for initiating proplatelet elongation in mature MKs. *Ex vivo*, MKs infused in the lung vasculature in the absence of ventilation or in the absence of oxygen have a reduced or abolished ability to produce platelets in the lung (40). *In vitro*, MKs exposed to increasing O_2_ levels strongly amplified thrombopoiesis by increasing the formation of proplatelets. High oxygen levels change intracellular reactive oxygen species (ROS) levels, resulting in enhanced mitochondrial fission, which in turn generates more mitochondrial ROS. ROS could contribute to the initiation of thrombopoiesis by the remodeling of the cytoskeleton for proplatelet and platelet release (76). Whether changes in oxygen concentration in the lung vasculature trigger a similar ROS-dependent mitochondrial fission and mitochondrial reactive oxygen species (mtROS) production remains to be studied *in vivo*.

### Interaction with lung stroma

Finally, the pulmonary circulation may also provide additional signals promoting platelet generation through specific lung endothelial cell–megakaryocyte interactions. Among the possibilities, Von Willebrand factor (vWF) on endothelial cell interaction with Glycoprotein 1b (GP1b) on MKs could be a mechanism for MKs to sense shear (77) and is an important regulator of proplatelet formation (78). vWF expression in endothelial cells is heterogeneous throughout the vascular tree, and its high level in lung endothelial cells could be another reason for efficient platelet production in the lung (79, 80). Altogether, the combinations of these important elements present in the lung vasculature may further increase platelet production, as suggested by *in vitro* experiments using microfluidic devices, where hydrodynamic shear and vWF anchoring improve proplatelet elongation and platelet production (74, 81). Nonetheless, platelet count is normal in mice lacking vWF, and it is reasonable to think that other pathways are involved in the interaction of MKs with the lung stroma. More work is needed to define them and understand their importance in platelet production.

## The lung environment modulates megakaryocytes and platelet functions

### Role of platelets in immune response and lung development and repair

It is becoming increasingly clear that platelets are involved in inflammation, infection, host response, and tissue development and regeneration by direct and indirect mechanisms. They can capture and engulf pathogens, but they also express adhesive molecules and many pro-inflammatory molecules (chemokines, cytokines, immune receptors, growth factor), interacting with various cells of the innate and adaptive immune system and initiating and modulating immune responses and tissue development and regeneration, including the lung (82–84). These functions have been previously reviewed (85–87). One would thus expect that patients with inherited (primary) or acquired (secondary) thrombocytopenia will be more prone to infection or to impaired tissue healing. These defects are not largely reported, and thrombocytopenic mice deficient for TPO receptor do not have apparent lung deficiencies. It is possible that studies would require continuous evaluation of the occurrence of infection or tissue damage, occasional events that may be hidden by the major defects in hemostasis. Another reason may be that as we observed in thrombocytopenic mouse models, only platelet counts severely decreased and combined with other specific defects in platelet activation, such as the absence of C-type lectin receptor 2 (CLEC-2) signaling, are associated with lung developmental defects (83). Platelets involved in immunity or repair may come from nonclassical pathways of megakaryopoiesis and may not be affected by impaired classical pathways such as TPO signaling. Some studies do provide clinical relevance of the role of platelets in infection and lung development. Infections are for instance common in patients with primary immune thrombocytopenia (ITP). A systemic review analyzing 13 studies show that adult patients with primary ITP have an increased risk of infection, from two to six times higher compared to the general population, and pulmonary infection is consistently reported as the primary site of infection (88, 89). If immunosuppressive therapies can explain this increased risk of infection, retrospective studies suggest that it is not the only reason, as patients with primary ITP had an increased risk of infection even before their diagnosis (90). Moreover, in another study, low platelet count is independently correlated with an increased risk of infection, even when adjusted for leukocyte count (91). In other contexts, a low platelet count is an independent risk factor of postoperative pneumonia in patients with acute aortic dissection (92), and during sepsis, admission thrombocytopenia is associated with enhanced mortality and a more disturbed host response independently of disease severity (93). A low platelet count is also observed and a risk factor for the development of bronchopulmonary dysplasia (BPD) in premature infants, characterized by the arrest of lung development, a decrease in the alveolar number, an increase in alveolar volume, simplification of the alveolar structure, and abnormal pulmonary microvascular morphology (94, 95). In the Hermansky–Pudlack syndrome, a genetic disorder with platelet dysfunction and dense granule defect, a high frequency of related pulmonary fibrosis is observed.

### MK heterogeneity—Immune MK populations

MK_L_ have a transcriptional profile suggesting a role in immunity and inflammation. Recent single-cell analyses revealed the existence of different MK populations exhibiting transcriptional heterogeneity in both humans and mice, suggesting specialized functions (96–98). These data suggest that the various functions of MKs are not fulfilled by the entire MK population as a whole but rather by distinct MK subpopulations with dedicated roles. Particularly, a population of MKs with lower ploidy and putative immune functions, resembling MK_L_, has also been identified in the bone marrow, liver, and spleen (13, 53, 96). Interestingly, reports suggest that distinct MK subpopulations are generated along distinct developmental routes, immune MKs being associated with the “emergency” route (49, 68, 98) (**Figure 1**). Alignment of different sets of MK Single cell (sc)-RNA-Seq data showed that subpopulations of BM MKs shared transcriptional similarity with MK_L_ gene profiles. After *L. monocytogenes* infection, one of the subpopulations achieved even greater similarity with MK_L_ than otherwise (53).

### MK plasticity and importance of the environment

Pariser et al. (20) have demonstrated that the immune phenotype of MKs is plastic and driven by the tissue immune environment, as evidenced by BM MKs having an MK_L_-like phenotype when adoptively transferred to the lung or under the influence of danger signals such as pathogen receptor challenge or Interleukin 33 (IL-33), a lung-associated danger signal, released upon cell damage. The important role of the lung microenvironment in MK_L_ development and specificity is also demonstrated by Yeung et al. (34) who showed that fetal MK_L_ still develop an immune phenotype, suggesting that the MK_L_ microenvironment before interactions with foreign antigens is able to promote the immune phenotype. The microenvironment is also important for MK final size and ploidy, as neonatal cells, usually smaller and with lower ploidy, are capable of producing adult‐sized MKs when placed in an adult microenvironment (99). If the lung niche supports the specialization of pulmonary MKs, one question remains: What is the dedicated role of this distinct population of immune MKs found in the lung?

### Role of the immune-MK population

**Figure 5:**
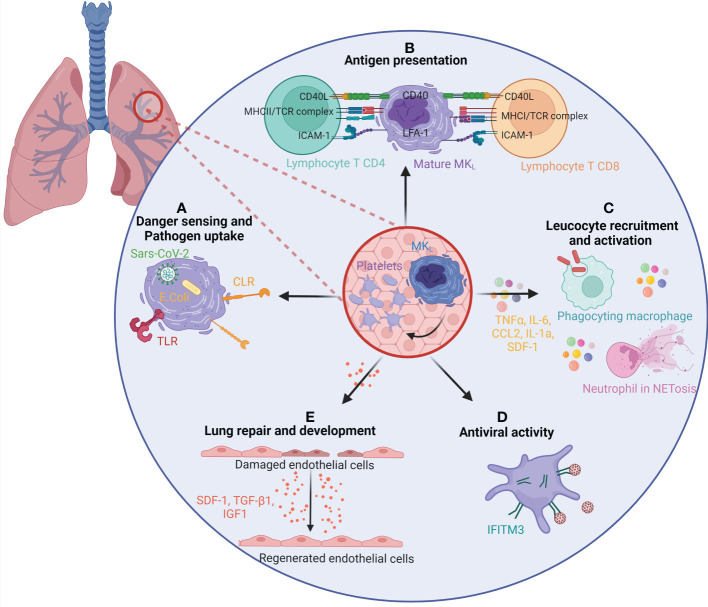
The role of lung megakaryocytes and platelets in immunity and repair. **(A)** MK_L_ and/or platelets present in the lung are able to sense and uptake pathogen through many different pathways. Pathogen-associated molecular patterns, such as Toll-like receptors (TLRs) or C-type lectin receptors (CLRs), present on MK_L_ surface can recognize the pathogens and uptake them. MK_L_ can also recognize internalized pathogens in their cytoplasm, such as SARS-CoV-2 virus or *E coli*. **(B)** MK_L_ and/or platelets can act as antigen-presenting cells for T CD8 and T CD4 lymphocytes. They uptake pathogens and antigens and present them on MHC class I and class II molecules. **(C)** The secretion of inflammatory molecules, such as TNFα, IL-6, CCL2, IL-1α, and SDF-1 confers an immune regulatory role to MKs and/or platelets. It enhances the migration and activation of leukocytes such as macrophages and neutrophils (bacterial uptake, NETosis). **(D)** The expression of interferon-stimulated genes, and particularly *ifitm3*, gives MK_L_ and platelets an antiviral activity. **(E)** MK_L_ and/or platelets participate in lung development and regeneration by the production of SDF-1, TGF-β1, and IGF1. CCL, chemokine (C-C motif) ligand; CD, Cluster of differentiation; IFITM3, Interferon-induced transmembrane protein 3; IGF1, Insulin-like growth factor 1; IL, Interleukin; MHC, Major Histocompatibility complex; MKL, Lung resident megakaryocytes; NET, Neutrophil Extracellular Traps; SDF-1/CXCL12, stromal cell-derived factor 1/ C-X-C motif chemokine 12; TCR, T cell receptor; TGF-β1, Transforming growth factor beta 1; TNF, Tumor Necrosis Factor.

The differential expressions of immune-regulatory genes in MK_L_ show a potential role in pathogen recognition and response, chemotaxis, myeloid cell activation, and response to type I interferon. These specific immune functions can be fulfilled by the MK itself, acting locally and independently of platelet production or transferred to its progeny and disseminated in the whole body through circulating platelets. MK immune potential has been previously reviewed (100), and we will focus here on immune roles validated by functional studies with MK_L_, spleen immune MK, or immune MK from BM sharing transcriptional identity with MK_L_, depicted in **Figure 5**.

#### Sensing danger/pathogen uptake

Compared to BM MK, MK_L_ express higher levels of pattern recognition receptor (Toll-like receptors (TLRs), C-type lectin receptors (CLRs)). They possess the ability to phagocytose or uptake bacteria *Escherichia coli in vitro* (20) and virus SARS-CoV-2 *in vivo* (49). Although MK_L_ and BM MKs both phagocytosed *E. coli*, MK_L_ internalized and digested more bacteria into phagolysosome. Interestingly, the virus is found in platelets from severe COVID-19 patients in phagolysosome-like structures suggesting virophagy (101), but platelets cannot be directly infected by the virus. In the case of SARS-CoV-2 infection, the virus, uptaken by the MK, may be transferred to its daughter platelets, participating in its dissemination (49).

#### Antigen processing and presentation

MKs can act as potent antigen-presenting cells to modulate T-cell responses *in vivo* by processing and presenting endogenous and exogenous antigens on major histocompatibility complex (MHC) class I molecules. Compared to BM MKs, MK_L_ express molecules like MHC class II (MHC-II) that are similar to tissue-resident leukocytes and Antigen Presenting Cell (APCs). Pariser et al. demonstrated *in vitro* and *in vivo* that MK_L_ internalized and processed both antigenic proteins and bacterial pathogens. Furthermore, MK_L_ induced CD4^+^ T-cell activation in an MHC II–dependent manner (20). In a similar manner, CXCR4^high^ MKs, an immune subpopulation of BM MKs resembling MK_L_, are able to present the ovalbumin antigens on their surface *via* MHC-I, activating CD8^+^ T cells (53). *In vitro* MKs have the ability to transfer MHC-I molecules loaded with foreign antigen to proplatelets (102). A similar immune MK population, found in the circulation and in the spleen, was actually shown to be the main APC in lupus responsible for TH17 polarization *via* MHC-II lupus antigen presentation and costimulatory molecule CD86 (103).

#### Immune/inflammatory molecule secretion and immune cell migration and activation

Compared to their BM counterpart, MK_L_ also express higher levels of inflammatory molecules such as chemokines, cytokines, or inflammatory lipids. At the protein level and in functional studies, lung MKs produced higher levels of inflammatory molecules [chemokine (C-X-C motif) ligand 1 (CXCL-1), Soluble Intercellular Adhesion Molecule 1 (sICAM-1), IL-1α, SDF-1, Macrophage Inflammatory Protein 3 (MIP-3), IL1RA, Tumor necrosis factor alpha (TNF-α), chemokine (C-C motif) ligand 2 (CCL-2)] after Lipopolysaccharide (LPS) stimulation compared with BM MKs (20). Wang et al. (53) showed that the immune MK subpopulation CXCR4^high^, but not CXCR4^low^ MKs, efficiently enhanced neutrophil and macrophage migration and bacterial phagocytosis in part through higher levels of TNF-α and IL-6 secretion. Another example of specific immune molecule expression is the generation of CD40L^hi^ platelets by MKs differentiated in the spleen of a mouse model of sepsis that has been involved in promoting the release of Neutrophil Extracellular Traps (NETs) from neutrophils, enabling efficient bacterial containment (13).

#### Antiviral

Response to viruses is among the ontology biological processes and signaling pathways enriched in MK_L_. Platelets have been involved in viral infection as reviewed previously (104). Among the genes significantly enriched, *ifitm3* is encountered in the three mouse MK_L_ studies and in circulating MKs during SARS-CoV-2 infection, as well as in platelets from infected patients (105). IFITM3 is an antiviral protein inhibiting viral cell membrane fusion and viral entry. Platelets and MKs upregulate IFITM3 (transcript and/or protein) upon infection in human (dengue, influenza, and SARS-CoV-2) and *in vitro*; overexpression of IFITM3 in MKs was sufficient to prevent dengue infection (106).

#### Lung development and repair

Another biological process enriched in MK_L_ is related to lung development and repair. MK_L_ and more particularly fetal MK_L_ are enriched in several growth factors and molecules critical in pulmonary alveolarization and development (*Tgfb1*, *fgf*, *sdf1*, *igf1*, *clec1b*). Fetal lung MKs may be a local source of growth factors that support the development of the embryonic lung (34). Platelet-derived CLEC2, encoded by *clec1b*, is required to separate blood/lymphatics in the developing lung interacting with lymphatic endothelial cells. Transforming growth factor beta (TGF‐β) production then drives the differentiation of myofibroblasts that are critical to primary septum formation and elastogenesis in alveolarization of the lung (83). Platelet-derived SDF-1 has been involved in lung regeneration after mouse lung pneumonectomy. SDF-1 stimulates the expression of SDF-1 receptors on pulmonary capillary endothelial cells, enhancing the proliferation of alveolar epithelial cells and lung regeneration (84). In adult lung, an excessive tissue repair response after injury can lead to fibrosis. In a mouse model of bleomycin-induced lung damage and fibrosis, pulmonary MKs have been involved in the proliferation of primary lung fibroblasts and transdifferentiation to myofibroblasts partially through the TGF-β1 pathway (56).

## Origin of resident megakaryocytes

One important remaining question is the ontogeny of MK_L_. When do they seed the lung and where are they coming from? Lung HSCs and hematopoietic progenitors (17, 19) have been identified in the adult lung. These cells migrate to the bone marrow and are able to reconstitute normal platelet count in thrombocytopenic mice. It is currently unknown if these progenitor cells can locally differentiate into MK_L_. MKs are less abundant but present in fetal lung before birth at embryonic day 13 in mouse, which is when the majority of murine hematopoietic development is occurring in the liver (34). They were also detected by Pariser et al. (20) in neonatal lung at birth (P0). Immune phenotypes of MK_L_ (MHC-II and ICAM1) were each expressed at much higher levels in adult MK_L_ compared with P0 mouse MK_L_, suggesting that the MK immune phenotype may be regulated by the postnatal environment. MK_L_ thus seed the lung before birth where they will acquire their specialization in contact to the lung environment. Fetal and neonatal megakaryopoiesis is characterized by rapid proliferation followed by full cytoplasmic maturation without polyploidization (107). Fetal MKs are smaller and with lower ploidy than adult MKs, but they are cytoplasmically mature, and they present characteristics of adult polyploid MKs, including the presence of granules, a well-developed demarcation membrane system, and proplatelet formation abilities (108). Thus, rather than immaturity, it has been suggested that fetal/neonatal megakaryopoiesis reflects a developmentally unique uncoupling of proliferation, polyploidization, and cytoplasmic maturation (107). This mechanism may allow fetuses and neonates to populate their rapidly expanding BM space and blood volume in this period of very rapid growth while maintaining normal platelet counts (109). Considering the shared properties between primitive MKs and MK_L_ (mainly diploid, bypass of classical progenitors, specialized functions in lung development or immunity), it is tempting to speculate that, similarly to tissue-resident macrophages, MK_L_ could differentiate from embryonic yolk sac-derived EMPs or MEMPs identified to colonize the organs during gestation (12, 110–112) (**Figure 1**). Clearly, questions remain to be fully addressed to determine the ontogeny, origin, and differentiation of MK_L_.

## MK identity and methodology advices for further studies

### Tool box for MK identification

The different subpopulations of MKs with specialized functions raise questions about MK identity and how to define an MK. The transcriptome profile of immune MKs has similarities with other myeloid cells. Are these cells really MKs with immune cell markers or could they be immune cells with MK markers or multipotential progenitors? Different techniques may be required to ensure the MK identity and are presented in **Table 2**. If the expression of specific markers such as CD41 is key in MK identification, it is important to make sure that platelets aggregated with leukocytes or other cell types are excluded. Platelets associate easily with other cells and will contaminate flow cytometry or RNA-Seq analysis of MKs identified or sorted as CD41^+^ cells. Results from analysis without attempt to exclude these aggregates should be interpreted with care. In mouse studies, the use of reporter mice expressing a nuclear-localized fluorescent protein can help in discriminating MKs from cells aggregated with platelets. The use of imaging flow cytometry is another way to differentiate MKs from leukocyte–platelet aggregates (114). It also adds morphological information using the shape, density, texture, and coincidence of fluorescence to discriminate different MK maturation steps (115). Morphology is an important parameter of MKs that can be obtained by immunohistology or immunofluorescence. Electron microscopy can be used to evaluate the identity and maturation status of MKs, the platelet-producing MKs usually presenting a large nucleus with a well-developed cytoplasm displaying demarcation membranes and numerous alpha-granules (113), as it has been shown for MKs retrieved from human pulmonary arteries (81). The ability of MKs to produce platelets is the final proof of MK identity and can be studied *in vitro* using flow chamber devices to mimic a more natural environment, but the dynamics of platelet formation can only be investigated since the development of two-photon microscopy (116). Reporter mice have been developed to visualize MKs *in vivo*, and their advantages and drawbacks are reported in **Table 3** (118–121). Regarding the ability of extravascular MK_L_ to produce platelets, they have been observed by intravital imaging, and these cells are sessile and have not been observed *in vivo* to release proplatelets/platelets during homeostasis (19). *In vitro*, Pariser et al. brought the evidence that isolated lung MKs were able to produce platelets after 3 days of culture. Wang et al. (53) also demonstrate the ability of an immune MK subpopulation identified in the BM (CXCR4^high^ MKs) to produce platelets, although it has relatively lower polyploidy, smaller cell size, and generated platelets in lower efficiencies compared to CXCR4^low^ MKs, suggesting that CXCR4^high^ MKs might be specialized for immune functions. The ability of MK_L_ to mature and to produce platelets *in vivo* during acute platelet need, inflammation, or infection remains however to be demonstrated.

**Table 2:** Tool box for MK identification.

Methods	Information	Disadvantages	References
Immuno-histology/ Immuno-fluorescence	• Identity and markers of maturation (CD41, CD42,CD61, vWF,…) • Cell and nucleus morphology • Presence of proplatelet/cytoplasmic elongation • Granule content staining • Cytoskeleton staining (Actin, tubulin, Vimentin, RhoA, VE-Cadherin) with subcellular localization • Interactions with other cells, stroma • MK/cm²	Fixed samples (dynamic evaluation impossible)	Rapkiewicz et al., 2020 (47) J. Balko, et al., 2022 (30) Valet et al., 2022 (13) Zhu et al., 2022 (49)
Electron microscopy	• Cell and nucleus morphology • Presence of demarcation membrane system • Granules	Fixed samples	Scandola et al., 2020 (113) Ouzegdouh et al., 2018 (81) Zhu et al.,2022 (49)
Flow cytometry	• High-throughput quantification of MK count/organ • Expression of membrane, cytoplasmic or nuclear protein allowing advanced population characterization (markers of maturation, inflammatory molecules, adhesion molecules, transcription factor,…) • Ploidy	Contamination with leukocyte-platelet aggregates	Pariser et al., 2021 (20) Lefrancais et al., 2017 (19), Yeung et al., 2020 (34)
Imaging flow cytometry	• Quantification of MK count/organ • Morphological information • High-throughput quantification of MK count/organ • Expression of membrane, cytoplasmic or nuclear protein allowing advanced population characterization • Ploidy • platelet-leukocyte aggregate discrimination	Complex analyses	McGrath, 2015 (114) Bush et al., 2021 (115)
Intravital microscopy	• Dynamic analysis of MK egress, proplatelet elongation, platelet formation • Interactions with other cells and stroma • Cell and nucleus morphology	Limited area Not in Human	Stegner & Heinze, 2020 (116)
Blood sampling	• Concentration of circulating MK/ml of blood	Dependent on blood sampling location, age, …	Pedersen, 1978 (37) Hume et al., 1964 (42) Pedersen & Cohn, 1981 (117)

CD, Cluster of differentiation; MK, megakaryocyte; Rho1, Ras homolog family member A; VE-cadherin, vascular endothelial cadherin; vWF, von willebrand factor.

**Table 3:** Comparison of MK/Platelet reporter mice.

Reporter mice	Information	Disadvantages	References
PF4-cre	• *Cxcl4* short promoter • *Cxcl4* is a strong driver • Lox insertion • No loss of native PF4 • Expressed early in MK maturation • Multiple choices of fluorochromes (including nucleus-targeted) • DTR-mediated deletion possible • Strong literature	• Expression in other hematopoietic lineages • Minor population of circulating leukocytes • Expression in a subset of monocytes/macrophages • Expression in immune cells under inflammatory conditions • Expression in epithelial cells of the colon • Alterations of the expression levels of the cytokine	F. Pertuy et al., 2014 (118); Gollomp and Poncz, 2019 (119)
CD41-YFP	• *GpIIb* locus • Plasma membrane localized • Upregulated during MK development	• Expressed in hematopoietic progenitors • No expression of cre • Only YFP available	Zhang et al., 2007 (120)
GP1ba-cre	• Endogenous GPIbα locus • Lox insertion • Expression specific to MK/platelet lineage • Multiple choices of fluorochromes • DTR-mediated deletion possible	• Might affect MK/platelet biology • No expression in developing MK - Later cre expression • Low cre level - Higher levels of proteins remaining after KO than in PF4-cre	Nagy et al., 2019 (121); Gollomp and Poncz, 2019 (119)

CD, Cluster of differentiation; CXCL4, chemokine (C-X-C motif) ligand 4; DTR, diphtheria toxin receptor; Gp, Glycoprotein; MK, megakaryocyte; PF4, Platelet Factor 4; YFP, Yellow fluorescent protein.

### Place and time of blood sampling for MK_circ_ quantification

The frequency of MKs in blood appears to depend upon the site in the body from which the blood is drawn. For example, blood from the inferior vena cava contains more MKs than the blood from the veins of the forearm (37). The cava drains an area rich in BM (pelvic girdle and spine), whereas the antecubital vein does not (bones of the forearm and hand). Moreover, with increasing age, bone marrow at several sites is replaced by adipose tissue. The primary sites of blood formation therefore gradually shift to the vertebrae, sternum, ribs, and pelvic bones. The number of circulating MKs in cubital venous blood declines during childhood: from 20 MK/ml in the first year of life to 5.5 MK/ml after the sixth year, reflecting the decrease in thrombopoiesis in the bone marrow of the fingers, hands, and forearms (117). Blood is almost always taken from antecubital veins and may be the reason why MKs are rarely observed in blood in normal conditions. The time of blood sampling may also be of importance. Traffic of hematopoietic cells and egress from the bone marrow are also impacted by biological rhythms. One study report that circulating MKs were found more frequently in the morning between 8:00 a.m. and 2:00 p.m (42). Circadian fluctuation was observed in platelet count and function (122), but more work is needed to understand if circulating MKs and platelet production in the lung are subjected to circadian oscillations.

## Concluding remarks

Research in recent years suggests that MKs and platelet production are found in various locations within the body, challenging historical models. Among extramedullary organs, the lung vasculature, where circulating MKs are trapped, has been proposed as an ideal site for the final step of platelet release, a phenomenon further increased when platelets are rapidly consumed such as in infectious disease, lung inflammation, excessive coagulation, or tissue damage. However, many unknowns remain regarding the signals that trigger MK release from the marrow in steady state and during emergency thrombopoiesis. It is also unknown whether the lung endothelium or its microenvironment locally promotes or modulates platelet production. MK subpopulations, including MK_L_, have been described and exhibit specialized functions in immune responses, stem cell maintenance, and tissue development and regeneration. Whether MKs in the pulmonary microcirculation and MK_L_ have other functions beyond their roles in the release of platelets is an emerging area of research. Challenging this cell identity crisis by ensuring quality control and multiple functional tests *in vitro* and *in vivo* is essential for future investigations. Finally, more studies are required to characterize the heterogeneity, plasticity, mobility, and roles of MKs according to their site of origin and development under basal conditions and disease.

## Author contributions

LG and LF designed the figures and EL wrote the manuscript. All authors contributed to the article and approved the submitted version.

## Funding

EL is supported by the Fonds de Recherche en Santé Respiratoire - Fondation du Souffle, the French National Research Agency (ANR JCJC), LG is supported by a PhD fellowship from the National Research Agency and LF is supported by a PhD fellowship from the Ministry of Research. Fondation du Souffle (Grant #198942) ANR JCJC (Grant #ANR-20-CE14-0045-01).

## Acknowledgments

Figures were created with Biorender.com. We thank Jean-Philippe Girard for proofreading of the manuscript.

## Conflict of interest

The authors declare that the research was conducted in the absence of any commercial or financial relationships that could be construed as a potential conflict of interest.

## Publisher’s note

All claims expressed in this article are solely those of the authors and do not necessarily represent those of their affiliated organizations, or those of the publisher, the editors and the reviewers. Any product that may be evaluated in this article, or claim that may be made by its manufacturer, is not guaranteed or endorsed by the publisher.
